# Generalized Pruritus and Gradual Loss of Vision as the Presenting Complaints of Acute HIV Infection: Management Challenges during COVID-19 Pandemic

**DOI:** 10.1155/2021/6436936

**Published:** 2021-12-01

**Authors:** Shrebash Paul, S. M. Mahbubur Rahman, Rajibur Rahman, Mahbubur Rahman, Quazi Mamtaz Uddin Ahmed, Rafiqul Alam, Fazle Rabbi Chowdhury

**Affiliations:** ^1^Department of Internal Medicine, Bangabandhu Sheikh Mujib Medical University, Dhaka, Bangladesh; ^2^Mahidol-Oxford Tropical Medicine Research Unit (MORU), Bangkok, Thailand

## Abstract

**Background:**

Although the prevalence of HIV is low in Bangladesh, there is a potential for an increased number of cases. This is because of high cross-border mobility (India and Myanmar) of people and increased injection drug abusers amongst youth in the cities and rural areas, HIV can present in many ways, from asymptomatic to advanced disease, including various atypical (generalized itching) and advanced (loss of vision) manifestations. A high degree of suspicion is required to diagnose HIV in a country like Bangladesh. Early diagnosis and prompt treatment are essential to have a better outcome.

**Methods:**

Here, we report two thought-provoking cases where patients were suffering from generalized itchy lesions (pruritic papular eruption) throughout the body for a long time and gradual loss of vision in another case.

**Results:**

Due to lack of suspicion, initially, HIV screening was not done. Both patients visited several health centres, but no diagnosis was made. Moreover, COVID-19 pandemic worsens the situation. Finally, they were diagnosed with HIV; unfortunately, one of them lost her vision due to CMV retinitis and another patient died of other complications.

**Conclusion:**

Ongoing COVID-19 pandemic put many challenges to ensure optimum care, especially for patients with long-sufferings like HIV. Clinicians have to have a very high degree of suspicion while dealing with patients presented with rare manifestations, particularly in a low endemic clinical setting.

## 1. Introduction

Bangladesh is still considered a low prevalent country for HIV/AIDS. The prevalence of HIV still remains very low (<0.1%) in the general population and a bit higher (<1%) in most at-risk populations [1, 2]. However, the country remains vulnerable to an epidemic because of the high prevalence in the neighbouring countries, risk behaviours amongst key populations (KPs), and high mobility of KPs across the country [1, 3]. The high cross-border mobility and increasing user of injection drug abusers could be a potential threat [1]. Acquired immunodeficiency syndrome (AIDS) is a fatal illness caused by a retrovirus known as the human immunodeficiency virus (HIV), which breaks down the body's immune system, leaving the victim vulnerable to a host of life-threatening opportunistic infections or neurological disorders or unusual malignancies [4,5]. HIV isolates are currently grouped into two types, HIV type 1 (HIV-1) and HIV type 2 (HIV-2). The global main agent of AIDS is HIV-1, while HIV-2 is restricted to some regions of Western and Central Africa [6]. Clinical manifestations of HIV/AIDS are diverse. The World Health Organization (WHO) recommended a universal clinical staging system for HIV/AIDS, which starts from asymptomatic infection up to severe and life-threatening opportunistic infection [7]. Here, we are reporting two interesting cases of acute HIV infection primarily presented with generalized itchy skin and gradual blindness.

## 2. Description of the Cases

### 2.1. Case 1

**Figure 1:**
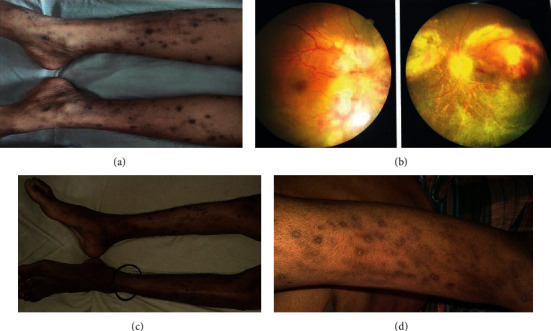
Clinical manifestations of the cases. (a) Multiple excoriated lichenified papules involving both lower limbs. (b) Disc swelling, haemorrhage, and exudates of both eyes suggesting retinitis. (c) Papular and pustular skin lesions involving lower limbs and (d) upper limbs.

A 45-year-old Bangladeshi woman was complaining (May 2020) of severe generalized itching and skin lesions over different parts of the body (extremities, neck, upper back, chest, oral cavity, and genitalia) for the last four years (Figure 1(a)). During the course of illness, she has taken multiple consultations and was prescribed different topical and systemic antifungals along with steroids. She also received a course of dapsone as a suspected case of dermatitis herpetiform, but showed no significant improvement of the condition. Her baseline investigations were inconclusive. She came to us while experiencing a gradual loss of vision of her both eyes for one month. She also had a history of recurrent fever and weight loss for the same duration. On query, she mentioned that her husband is also suffering from a similar skin lesion for a long time and residing in South Africa for the last 08 years. The rest of her family members are in good health; there was no history of similar lesions in her family. She denied any history of tuberculosis or contact with smear-positive tuberculosis patients. She did not receive blood transfusion in the past.

She was found emaciated, moderately anaemic, normal vitals, and without any lymphadenopathy. On skin survey, she was found to have multiple excoriated lichenified papules over different parts of the body, e.g., extremities (Figure 1(a)), neck, and upper back. There were also exudation and crusting in some lesions. There were some whitish patches over her tongue and oral cavity and a nonhealing ulcer present over her labia. Fundoscopy reveals disc swelling, haemorrhage, and exudate present over superior and inferior quadrants of the right eye and a pale disc with exudates in the superior and inferior retinal quadrants of the left eye.

Routine investigations, including ultrasound abdomen, were normal. Haematological and biochemical profiles are given in Table 1. The fundal photograph was taken and showed disc swelling and splinter haemorrhage, exudate present over superior and inferior quadrants in the right eye, and pale disc with vascular sheathing, superiorly and inferiorly retinal exudate present in the left eye (Figure 1(b)). Recurrent, severely pruritic and exudative papular eruptions, which were refractory to treatment, along with ophthalmic findings, led to getting her investigated for HIV. The enzyme-linked immunosorbent assay (ELISA) for HIV came out to be positive. Other investigations including the serum venereal disease research laboratory (VDRL) test, TPHA, serum hepatitis B surface antigen (HBsAg), antibody against HCV, and antibodies (IgM + IgG) against HSV 1 and 2 were negative. X-ray of chest and ultrasonogram (USG) of the abdomen were normal.

The ELISA for HIV came out to be positive. Anticytomegalovirus (CMV) IgG was positive. The patient's CD4 count was 237 cells/mm. She was labelled as a case of HIV/AIDS, WHO stage 3, and she was started tenofovir, lamivudine, and efavirenz (TDF + 3 TC + EFV) regimen immediately. Due to COVID-19 pandemic, advanced ophthalmological services were limited and took a long time for consultation. Injection ganciclovir was advised to the patient, but she could not afford it. The patient clinically improved during discharge with impending concerns regarding her visual impairment.

### 2.2. Case 2

A 19-year-old male patient was admitted (August 2020) with complaints of severe generalized papular and pustular skin lesions over different parts of the body (extremities, face, upper back, and chest) for the last two years (Figures 1(c) and 1(d)). He consulted multiple physicians and received multiple antibiotics/antifungals like the previous case without any significant clinical improvement of the health condition. Later, he developed fever for two months, a productive cough with mucopurulent sputum, with a history of significant weight loss. He also experienced chronic diarrhoea in recent times.

He had a history of addiction to cannabis and amphetamine. However, he denies any history of injection drug abuse and unprotected sexual exposure. He did not give any history of contact with smear-positive pulmonary tuberculosis patients or blood transfusion. All of his family members were in good health. On examination, the patient was cachectic, moderately anaemic, vitals within normal limits except temperature (102°F), and no lymphadenopathy. There were generalized papulopustular lesions involving extremities, face and upper back, chest with exudation, and a few bullous lesions over the forehead. He also had oral candidiasis and mild hepatomegaly.

Laboratory profiles are given in Table 1. An ELISA for HIV was found positive. He was diagnosed with WHO clinical stage 2 HIV/AIDS. Viral load and CD4 count were not done as the virological service in this regard was shut down due to COVID-19 pandemic. He was put on TDF + 3TC + EFV regimen. He was well, discharged, and referred to a divisional hospital (his hometown, 250 km away from Dhaka) for regular follow-up and management. On telephonic follow-up, we came to know that he was stable for three months and then again developed high fever and progressive loss of consciousness. We advised to admit to hospital; however, due to COVID-19 crisis, long-distance travel was limited. He was admitted to a local hospital and unfortunately died after three days of illness.

## 3. Discussion

Early detection and treatment are the keys to success in clinical management of HIV/AIDS patients. Bangladesh is still at the beginning of an evolving epidemic of HIV-associated disease. There are 28 HIV testing and counselling (HTC) centres and 103 NGO-operated HTS (HIV testing services) centres in different tertiary care hospitals and district hospitals (in 23 HIV priority districts). These recentres are equipped with a full laboratory facility to confirm the diagnosis of HIV and for proper pre and posttesting counselling. Treatment of HIV/AIDS in Bangladesh is primarily based on nonnucleoside reverse transcriptase inhibitor regimes with limited availability of protease inhibitors (PI). The Government of Bangladesh supplies drugs for the treatment of HIV/AIDS at free of cost.

Dermatological involvement in HIV/AIDS is not uncommon. Its prevalence ranges 11–46%, more common in less developed countries [8]. Amongst the dermatologic manifestations, pruritic papular eruptions (PPEs) are the most common. PPEs can be the primary presentation of HIV/AIDs in some cases [8, 9]. PPEs are characterized by longstanding bilaterally symmetric pruritic papules and sterile pustules on the trunk and extremities in an HIV-infected patient. Though the exact aetiology of PPEs is not known yet, an altered and exaggerated immune reaction to arthropod antigens might be responsible [10]. Follicular (eosinophilic, Demodex, staphylococcal, and pityrosporum folliculitis) and nonfollicular pruritic eruptions, including scabies, insect bites, prurigo nodularis, and eczematous eruptions, are the differential diagnosis of PPEs [11].

Liautaud et al. had shown in one study that in 79% of HIV/AIDS patients, intensely pruritic eruptions were the first markers of HIV. They found that the eruptions appeared a mean of 8 months earlier than the diagnosis of either Kaposi's sarcoma or opportunistic infection [10]. Thus, PPEs are acting as the indicators of advancing immunosuppression. However, due to lack of awareness, clinicians can often miss it. It delays the diagnosis and can lead to morbidity as happened in this case.

Management of HIV/AIDS patients presented with dermatological problems primarily depends on antiretroviral therapy. In the majority of the cases, treatment of dermatological diseases in HIV/AIDS patients is similar to that of HIV-negative patients. Frequent follow-up is required for patients who are given prolonged high-dose systemic steroids because of the immunosuppressive effects. Sometimes, phototherapy can be used to alleviate pruritus along with other antipruritic treatments [12].

Ophthalmological involvement in HIV/AIDS is also not uncommon. Eye involvement can occur in up to 70% of HIV patients [13]. The spectrum of ophthalmic manifestations is vast and wide, and it ranges from simple blepharitis to complete blindness [13–15]. Opportunistic infection of the retina is a grievous complication of HIV/AIDS.

CMV retinitis is the most common intraocular infection in patients with AIDS, and it represents about 90% of the infectious retinitis in HIV patients. CMV retinitis may present in 20–30% of all HIV/AIDS patients [13, 14]. It causes the highest number of AIDS-related ophthalmological morbidity. Delayed treatment or untreated CMV retinitis ultimately terminated to blindness, which is potentially irreversible. Diagnosis of CMV retinitis is clinical; therefore, a high degree of clinical suspicion is required. It usually indicates an advanced stage of disease and occurs in patients with low CD4 count during the late stages of the disease. Along with ART, CMV retinitis needs systemic or local (intravitreal) antivirals like ganciclovir, foscarnet, or cidofovir therapy [16]. Close ophthalmological observation and CD_4_ count monitoring are essential. Here, we report a case of CMV retinitis; however, reports of acute CMV have previously been described in primary HIV infection [17].

The advent of highly active antiretroviral therapy (HAART) brings a revolution in the natural history of HIV/AIDs by significantly reducing morbidity and mortality. Delayed diagnosis of HIV infection is a burning public health problem. Delay in diagnosis causes the increasing number of opportunistic infections and malignancies as well as increased transmission of the infection in the community [18, 19].

Delayed diagnosis of dermatological disease in HIV/AIDS patients causes increased rates of different severe skin infections and malignancy like Kaposi's sarcoma [20]. Delayed diagnosis of CMV retinitis is a major cause of blindness in HIV/AIDs patients, as in our case.

In both cases, the cause of delayed diagnosis was lack of suspicion, as the prevalence of HIV in Bangladesh is low. However, there is always a potentiality of increase in this infection because of cross-border mobility and higher usage of drugs through common syringes amongst the youth. In the first case, the patients' husband lives in South Africa (a high HIV endemic country) and have a similar problem, which was a striking clue. For the second case, patient's personal behaviour (drug abuser) and later development of classical clinical presentations (fever, secondary infections, weight loss, and diarrhoea) helped to confirm the diagnosis.

Total services for HIV/AIDS patients are maintained by a continuous and step by step mechanism. COVID-19 disrupted this chain of services for the HIV/AIDS patient. As a result, diagnostic kits and antiretroviral drugs became limited for HIV/AIDS patients across the globe. Service disruptions associated with COVID-19 are affecting global efforts to end the epidemics of HIV. The ongoing COVID-19 crisis in Bangladesh seriously impacted the treatment of non-COVID patients. We faced enormous challenges, delay to ensure optimum clinical management (acute medicine and ocular care) for both the cases and eventually lost one of the patients. The pandemic has already brought a negative impact on HIV treatment services, access to treatment, and adherence throughout the world [21]. Bangladesh is not an exception.

While managing cases of refractory generalized, pruritic papular lesions, HIV should be considered in the differential diagnosis. PPEs are indicators of underlying advancing immunosuppression and had a significant influence on the patient's quality of life. Ophthalmic manifestations seek urgent screening for CMV because the delayed diagnosis may cause loss of vision.

## Figures and Tables

**Table 1:** Haematological and biochemical profile of the patients.

Test	Case 1	Case 2	Normal range
Haemoglobin	8.8 gm/dl	7.8 gm/dl	13–17 gm/dl
Total WBC count	4.5 × 10^3^/*µ*L	2.12 × 10^3^/*µ*L	4–11 × 10^3^/*µ*L
Total platelets count	300 × 10^9^/*µ*L	190 × 10^9^/*µ*L	150–450 × 10^9^/*µ*L
Alanine transaminase (ALT)	80 U/L	106 U/L	Up to 45 U/L
Aspartate aminotransferase	72 U/L		Up to 36 U/L
Alkaline phosphatase	178 U/L		Up to 100 U/L
Total bilirubin	0.7 mg/dL		0.2–1.1 mg/dl
HbA1c	5.4%	4.0%	4.5–6.3%
S. creatinine	0.54 mg/dl	1.31 mg/dl	0.6–1.3 mg/dl
Hepatitis B surface antigen	Negative	Negative	N/A
Hepatitis C antibody screen	Negative	Negative	N/A
HSV 1 + 2 antibody (IgG + IgM)	Negative		N/A
Anti-CMV IgM	Negative		N/A
Anti-CMV IgG	Positive		N/A
VDRL and TPHA	Negative		N/A
Sputum for AFB, Gene Xpert, and fungus		Negative	
Mantoux test (MT)		2 mm	Up to 10 mm
Chest X-ray	Normal	Normal	
Blood and urine culture		No growth	
Pus culture from the skin		Methicillin-resistant *Staphylococcus aureus* (MRSA)	

## Data Availability

The data used to support the findings of this study are available from the corresponding author upon request.
